# Metabolic Characteristics of Schisantherin B in Mice with Metabolic-Associated Fatty Liver Disease

**DOI:** 10.3390/metabo15120763

**Published:** 2025-11-25

**Authors:** Fei-Long Liu, Zhao-Rui Song, Meng Gao, Xi-Yuan Feng, Meng-Yang Wang, Zhi-Hong Zhang, He Li, Chun-Mei Wang, Jing-Hui Sun

**Affiliations:** School of Pharmacy, Beihua University, Jilin 132013, China; m18831442942@163.com (F.-L.L.); 18686408425@163.com (Z.-R.S.); s9912062636@163.com (M.G.); 18004369287@163.com (X.-Y.F.); 15643584357@163.com (M.-Y.W.); zhzhang518@163.com (Z.-H.Z.); yitonglh@126.com (H.L.); wangcm74@126.com (C.-M.W.)

**Keywords:** schisantherin B, metabolic-associated fatty liver disease, CYP450, pharmacokinetic

## Abstract

**Objectives**: We aimed to observe the pharmacokinetic differences of schisantherin B (STB) in the blood and liver of normal and metabolic-associated fatty liver disease (MAFLD) mice, as well as the changes in CYP450 enzymes in MAFLD mice. **Methods**: A MAFLD model was established in C57 mice fed a high-fat diet. Blood and liver samples from mice administered STB (5 mg/kg) were analyzed by high-performance liquid chromatography–electrospray tandem mass spectrometry (HPLC-ESI-MS) to identify major metabolites of STB and assess the activity of CYP450 enzymes. Pharmacokinetic parameters were calculated using DAS 3.0 software. The cocktail assay method was employed to determine CYP450 enzyme activity in hepatocytes in vitro. **Results**: The activities of CYP1A2, CYP2B6, CYP2C9, and CYP3A4 were significantly decreased, while the CYP2E1 activity was significantly increased in MAFLD hepatocyte model. In vitro liver microsomal experiments revealed that STB was primarily metabolized by CYP3A4 and CYP2C9. Compared to normal mice, STB in the liver tissue of MAFLD mice showed a significantly reduced area under the curve (*AUC*) and peak concentration (*C_max_*), prolonged half-life (*t*_1/2_), decreased mean retention time (*MRT*), and increased clearance (*CL*). In contrast, the *AUC*, *C_max_*, and *t*_1/2_ of STB in the serum of MAFLD mice were significantly increased, while the *CL* was decreased. **Conclusions**: Changes in the activity of liver microsomal enzymes following fatty liver injuries in MAFLD mice may lead to pharmacokinetic differences in STB, thereby affecting its metabolism in the liver.

## 1. Introduction

Metabolic-associated fatty liver disease (MAFLD) refers to a fatty liver disease accompanied by one or more cardiovascular and metabolic risk factors, in the absence of excessive alcohol consumption, affecting approximately 25% of the general population globally and over half of patients with metabolic disorders [1]. MAFLD has become one of the most significant causes of liver diseases worldwide and may soon emerge as a primary driver of liver diseases progressing to end-stage liver diseases [2]. Its pathogenesis is driven by metabolic inflammation (meta-inflammation), a chronic low-grade inflammatory state originating from adipose tissue dysfunction and hepatocyte lipotoxicity, ultimately leading to progressive liver injury [3].

Cytochrome P450 (CYP) enzymes are a superfamily of heme-dependent monooxygenases widely distributed in organisms, with the highest expression in the liver. In humans, 57 putatively functional genes have been identified, approximately 12 of which are crucial for the metabolism of most clinically used drugs [4]. The expression and activity of these enzymes exhibit significant variabilities both between and within individuals. Key determinants of interindividual differences include genetic polymorphisms, sex, age, disease status, and environmental factors [5]. As the core enzyme system of drug metabolism, CYP is involved in approximately 75% of clinical drug oxidative metabolism [6]. Recent studies have revealed that CYP activities are closely associated with the pathogenesis of metabolic diseases. The dysregulation of hepatic lipid metabolism can significantly alter CYP expression and function: on one hand, subtypes such as CYP2E1 and CYP4A are overactivated to promote lipid peroxidation and reactive oxygen species (ROS) production, thereby exacerbating liver injuries [7]; on the other hand, the activity of drug-metabolizing enzymes like CYP3A4 and CYP2C19 is inhibited, leading to reduced drug clearance and increased medication risks for patients with MAFLD [8].

*Schisandra chinensis* (Turcz.) Baill. is a dried mature fruit of the Magnoliaceae plant. As a traditional Chinese medicine, its medicinal history covers thousands of years [9]. *Schisandra chinensis* is commonly used in clinical practice to treat chemical and viral liver injuries. The chemical composition of *Schisandra chinensis* is relatively complex, with the main active ingredients including lignans and polysaccharides. Studies have shown that lignans in *Schisandra chinensis* can significantly delay the progression of MAFLD [10,11,12].

**Figure 1:**
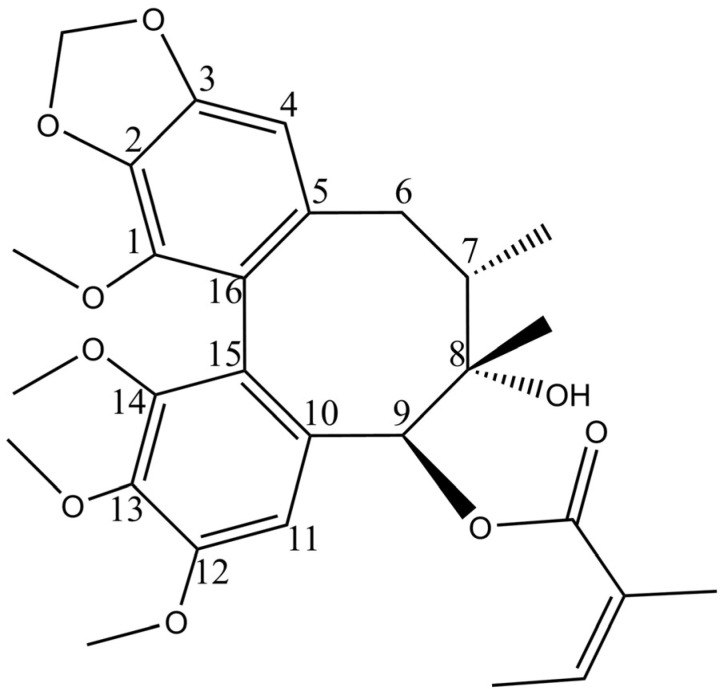
Chemical formula of STB.

Our previous studies showed that schisantherin B (STB, Figure 1) could regulate various indicators in the serum of hyperlipidemic mice, including reducing the levels of total cholesterol (TC), triglycerides (TG), and low-density lipoprotein cholesterol (LDL-C) and increasing the levels of high-density lipoprotein cholesterol (HDL-C). STB can correct blood lipid disorders and alleviate liver steatosis, indicating that it is one of the effective ingredients in *Schisandra chinensis* for the treatment of MAFLD, with a good research value [13].

Liver enzymes play a crucial role in drug metabolism in the body. Liver disease patients often have pathological changes in the liver, and the pharmacokinetics of drugs may also change accordingly [14]. It has been shown that the activity of drug-metabolizing enzymes may change in chronic liver diseases, which may be due to their affecting the clearance of drugs through liver metabolism or bile excretion, thereby affecting drug metabolism and excretion processes [15]; so, it is crucial to jointly study the pharmacokinetics of drugs in the body and the changes in liver enzyme activity. Our research group also studied the pharmacokinetic characteristics of *Schisandra chinensis* lignans in vivo and their distribution in tissues [16,17]. So far, there have been no studies on the pharmacokinetic differences of STB in normal and MAFLD models in vivo.

Therefore, in this study, HPLC-ESI-MS/MS was used to determine the drug concentrations in the blood and liver of normal and MAFLD mice after oral administration of STB, and the pharmacokinetic characteristics were compared and analyzed. The cocktail method was also used to determine the activity of various subtypes of CYP450 enzymes in MAFLD liver cells. Simultaneously, metabolomics methods were used to conduct in vivo metabolic analysis on blood and liver samples, in order to provide references for the clinical application of *Schisandra chinensis*.

## 2. Materials and Methods

### 2.1. Drugs and Reagents

Schisantherin B was purchased from Chengdu Pufide Biotechnology (purity more than 99.8%, Chengdu, China); phenacetin, coumarin, diclofenac, chlorzoxazone, bupropion, testosterone, oleic acid, palmitic acid, α-naphthoflavone, pilocarpine, ticlopidine, sulfaphenazole, quinidine, sodium diethyldithiocarbamate, and ketoconazole were purchased from MACKLIN (Shanghai, China). Dexmedetomidine reference standard was purchased from TMstandard (Jiangsu, China); AST, ALT, TG, TC, LDL-C, and HDL-C detection kits were purchased from Nanjing Institute of Bioengineering (Nanjing, China). NADPH and 0.1 M PBS buffer were purchased from IPHASE (Beijing, China); pancreatic enzymes (tyrisin) and streptomycin were purchased from Beijing Dingguo Changsheng Biotechnology (Beijing, China). Fetal bovine serum and DMEM/F12 culture medium were purchased from Shanghai Daxier Biotechnology (Shanghai, China); acetonitrile and methanol were purchased from SHIELD (chromatographic grade, Tianjin, China). The basic feed and high-fat feed for mice were purchased from Changchun Yisi Experimental Animal Technology Co., Ltd. (Changchun, China); the components of the high-fat feed were as follows: lard (15%), sucrose (20%), cholesterol (1.2%), sodium cholate (0.2%), casein (10%), calcium hydrogen phosphate (0.6%), and basic feed (53%) [18].

### 2.2. Animal Grouping and Administration

Four-week-old male C57 mice were purchased from Changchun Yisi Experimental Animal Technology Co., Ltd. [body weight: 18–20 g, license number: SCXK (Ji) 2023-0002, Changchu, China]. The temperature in the animal room was kept at 20–22 °C, the humidity was about 50%, and the natural day–night cycle was maintained. This research protocol was approved by the Institutional Animal Care and Use Committee (IACUC) of Beihua University (No. 2025051202), and all experimental procedures were carried out in accordance with the “Regulations on the Management of Experimental Animals”.

Seventy-four mice were randomly divided into a control group and a model group. Mice in the control group (Control, CON) were fed a normal diet, while those on the MAFLD model group (Model, MOD) were fed the high-fat diet (HFD). After 16 weeks, 10 mice were randomly selected from each group and anesthetized by the intraperitoneal injection of 0.3% pentobarbital sodium (50 mg/kg). The blood samples were collected and centrifuged at 3000 r/min for 10 min to separate the serum, and the serum standard curves were drawn for methodological investigations. The levels of AST, ALT, TC, TG, LDL-C, and HDL-C in the serum were measured using the detection kits. The liver tissue of mice was fixed in a 10% formalin solution, and then subjected to ethanol dehydration, paraffin embedding, slicing, and dewaxing treatment in sequence. Hematoxylin & eosin staining was performed, and the pathological changes in the liver tissue were observed under an optical microscope (×100 and ×200).

### 2.3. Preparation of Serum and Liver Samples for Pharmacokinetic Studies

The remaining mice in the control group and model group were given carboxymethyl cellulose sodium solution containing STB (5 mg/kg) by gavage. At 9 time points (0.5, 1.0, 1.5, 2.0, 3.0, 4.0, 8.0, 12.0, 24.0 h) after administration, 3 mice were taken from each group, and their serum and liver tissue were collected. Two hundred μL of the serum sample was added with twice the volume of methanol; then, the serum–ethanol solution was vortexed for 30 s and centrifuged at 13,000 r/min for 5 min to obtain the supernatant. The supernatant was transferred to a new EP tube and concentrated in a nitrogen dryer until dry; then, 1.5 mL of methanol was added with for redissolving, and the redissolved sample solution was mixed by vortex for 30 s. The sample solution was filtered through a 0.45 μm filter membrane. The filtrate was placed in a sample bottle, and the bottle was kept at 4 °C for use. One gram of mouse liver tissue was washed with pre-cooled normal saline, and the saline on the surface was absorbed until dry with a piece of filter paper. The liver tissue was prepared into its homogenate by adding 4 mL of normal saline in an ice bath, and the subsequent preparation was the same as that for the serum samples.

### 2.4. Cell Modeling

Oleic acid (OA) and palmitic acid (PA) are the two most commonly used free fatty acids in constructing a cell model of metabolic-associated fatty liver disease (MAFLD), which can simulate the pathological process of lipid accumulation in liver cells [19]. AML-12 cells were selected for culture in this experiment. A palm acid solution at a final concentration of 3.3 mM was prepared with 100 mM NaOH, 10% BSA, and palm acid, and an oleic acid solution at a final concentration of 6.6 mM was prepared with 10% BSA and oleic acid. The solutions were divided into centrifuge tubes after filtration and stored at 4 °C for use. The cells were cultured in the complete culture medium added with 10% free fatty acids (palmitic acid v:oleic acid v = 1:1) until obvious lipid droplets appeared in the cells, indicating a successful modeling.

#### 2.4.1. Cocktail Probe [20]

The probe substrate was added to DMSO to prepare a mixed mother liquor containing 200 μM phenacetin, 20 μM coumarin, 120 μM chlorzoxazone, 40 μM testosterone, 20 μM dexmedetomidine, 200 μM bupropion, and 40 μM diclofenac. The mixed mother liquor was packed and stored at −80 °C for use.

#### 2.4.2. In Vitro Incubation of Liver Cells

Referring to the methods proposed by Feidt [21] and Hewitt [22], the viability of liver cells was determined using trypan blue. Normal liver cells with viability more than 85% and model liver cells were selected for counting, with a concentration of 2 × 10^5^ cells/cm^2^. The cells were seeded onto 24-well plates, and 500 μL of Williams medium was added into each well. The cells were cultured at 37 °C and in 5% CO_2_ for 24 h to allow them to adhere to the walls. The old culture medium was discarded before incubation. The cells were washed with PBS twice, and then, 500 μL of incubation medium (475 μL serum-free medium, 24 μL NADPH, and 1 μL mixed probe substrate) was added. The cells were incubated at 37 °C and in 5% CO_2_ for 30 min, and then, 500 μL of ice cold acetonitrile (IS: reserpine 50 ng/mL) was added to terminate the reaction. The final volume of organic solvents in the incubation system was less than 1%. The cell solution was centrifuged at 13,000× *g* and 4 °C for 10 min, and then the supernatant was collected and stored at −20 °C for analysis on LC-MS.

#### 2.4.3. In Vitro Incubation of Liver Microsomes

The residual blood on the surface of normal mouse liver was washed away with 0.1 mol/L PBS buffer (pH 7.4), and the gallbladder was removed. The liver tissue was prepared into its homogenate by adding buffer solution in a 1:4 ratio in an ice bath. The homogenate was centrifuged at 10,000× *g* and 4 °C for 30 min to obtain the supernatant. The supernatant was centrifuged at 105,000× *g* and 4 °C for 60 min, and the precipitate was taken out and suspended in 0.1 mol/L PBS-20% glycerol to obtain liver microsomes. The liver microsomes with a final concentration of 0.5 g/mL were added to the NADPH regeneration system, washed with 0.1 M PBS, and preincubated at 37 °C for 5 min. Then, 7 inhibitors (Table 1) and schisantherin B at a final concentration of 50 mmol were added to the system, and the sample solution was incubated at 37 °C for 30 min, followed by adding ice acetonitrile containing an internal standard to terminate the reaction, and the sample solution was centrifuged to collect the supernatant for LC-MS analysis.

### 2.5. HPLC-ESI-MS Analysis

#### 2.5.1. HPLC-ESI-MS Conditions

An Ultimate 3000 ultra-high performance liquid chromatography system (Thermo, San Jose, CA, USA) combined with a Supelco C18 column (3.0 × 50 mm, 2.7 μm, Sigma-Aldrich, St. Louis, MO, USA) were used for chromatographic separation. The column temperature was maintained at 35 °C; mobile phase A was acetonitrile and B was water, and both of them contained 0.1% formic acid. The metabolite gradient elution procedures are shown in Table 2: the injection volume was 5 μL, and the flow rate was 0.4 mL/min. The gradient elution procedures for probe substrates and specific metabolites are shown in Table 3: the injection volume was 5 μL, and the flow rate was 0.4 mL/min.

A Q-Orbitrap MS/MS system (Thermo, San Jose, CA, USA) equipped with an electric spray ion source (positive and negative ion mode) was used for the mass spectrometry detection. Ion source parameters: the sheath gas flow rate was 40 Arb, the auxiliary gas flow rate was 10 Arb, the purge gas flow rate was 1 Arb, the capillary voltage was +4.0 kV, and the capillary temperature was 350 °C. Full scan mass spectrometry conditions: central cutting mode, resolution of 70,000, scanning range *m*/*z* 150–2000 Da. The automatic gain control (AGC) target value was 1 × 10^6^, with a maximum injection time of 100 ms. Full MS/ddMS^2^ mode was adopted in the secondary mass spectrometry: the resolution was 17,500, the AGC target value was 1 × 10^5^, the maximum injection time was 50 ms, and the normalized collision energy (NCE) was 25–45 eV. The chromatographic parameters of the probe substrates are shown in Table 4.

#### 2.5.2. Calibration Curve, LOQ, and LOD

STB and schisantherin B (internal standard, IS) were well mixed with 200 μL of the serum of blank control mice (liver tissue homogenate), and an appropriate amount of methanol was added to the mixture for ultrasonic dissolution to obtain the standard stock solution. The standard stock solution was diluted to prepare sample solutions with concentrations of 6, 3, 1.5, 0.8, 0.4, 0.1, 0.05, 0.02, 0.01, 0.005, and 0.002 mg/mL. After filtration through a 0.45 μm organic membrane, the sample solutions were measured according to the above LC-MS conditions, and the chromatographic peak areas of STB and IS were recorded. An internal standard curve was drawn by plotting the relationship between the peak area of STB/IS and the concentration of STB, in which the quantification limit was set at a signal-to-noise ratio of 10:1, and the detection limit was set at a signal-to-noise ratio of 3:1.

#### 2.5.3. Precision and Stability

The standard solution with a concentration of 1.5 mg/mL was continuously injected three times, with 10 μL each time, and the relative standard deviation (RSD) was calculated based on the peak area obtained to evaluate the precision of the experimental method. The above samples were measure at time points of 1, 2, 6, 12, and 24 h, and RSD values of the peak area at different time points were calculated to investigate the stability of the solution.

### 2.6. Data Analysis

DAS 3.0 software was used for non-compartmental analysis to calculate the pharmacokinetic parameters, and the results were expressed as the mean ± standard deviation (mean ± SD). The serum biochemical indicators and major pharmacokinetic parameters between the model group and the control group were analyzed using SPSS 20.0 software, in which the *t*-test was used for independent samples, and *p* < 0.05 indicated statistical significance. Enzymatic kinetic parameters were analyzed using GraphPad Prism 8. Metabolites were identified and analyzed using Compound Discoverer 3.3, Mass Frontier 8.0, and SIMCA 14.1.

## 3. Results

### 3.1. Serum Biochemical Indicators and Histomorphological Changes in Liver Tissue

**Figure 2:**
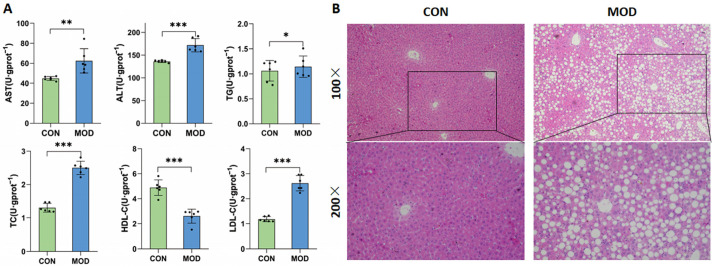
Results of serum AST, ALT, TC, TG, LDL-C, and HDL-C levels (**A**) and morphological changes in the liver tissue (**B**) of mice (mean ± SD, *n* = 3). * *p* ˂ 0.05, ** *p* ˂ 0.01, *** *p* ˂ 0.001.

As shown in Figure 2, the levels of AST, ALT, TC, TG, and LDL-C in the serum of mice in the model group were significantly increased, while the level of HDL-C was significantly decreased compared with the control group (*p* ˂ 0.05). The liver pathology results showed the significant lipid degeneration, inflammatory infiltration, and lipid vacuoles in the liver tissue, indicating the successful establishment of the MAFLD model.

### 3.2. In Vivo Pharmacokinetic Parameters of STB

**Figure 3:**
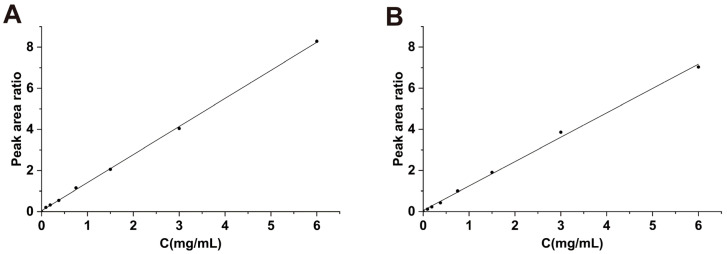
Blank serum standard curve (**A**) and blank liver tissue standard curve (**B**).

The regression equation for the standard curve of the serum STB was y = 1.365x + 0.0471, and R^2^ = 0.9996, with a linear range of 0.005–6 mg/mL. The regression equation for the standard curve of the liver tissue homogenate was y = 1.1859x + 0.058, and R^2^ = 0.9977, with a linear range of 0.005–6 mg/mL (Figure 3). In this study, the quantification limit of STB was determined to be 0.005 mg/mL, the detection limit was 0.002 mg/mL, the precision RSD value was 0.83–2.06%, the stability of the RSD values was 0.43–1.29%, the repeatability of the RSD values was 0.53–1.79%, and the average recovery rate was 92.7–98.5%. The above results indicate that the HPLC analysis method established in this study is accurate and reliable.

**Figure 4:**
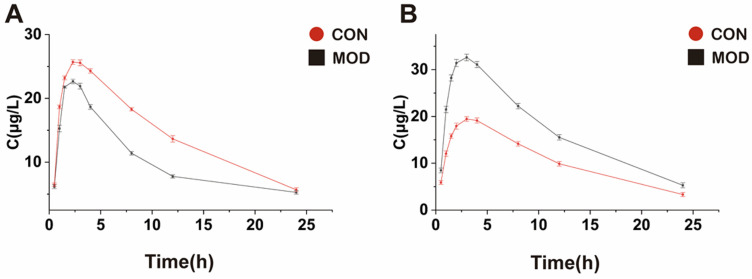
Serum pharmacokinetic curve (**A**) and liver tissue pharmacokinetic curve (**B**) (*n* = 3).

The peak area results of each time period detected in LC-MS was substituted into the standard curve to obtain the corresponding concentration values of STB. The concentration–time curves of STB in serum and liver are shown in Figure 4. The pharmacokinetic test results showed that the *AUC* of STB was reduced, the *C_max_* was decreased, *t*_1/2_ was prolonged, the *T_max_* and *MRT* were shortened, and the *CL* was increased significantly in the liver tissue of mice in the MAFLD model group compared with the control group. The *AUC* of STB was increased, the *C_max_* was increased, the *t*_1/2_, *T_max_*, and *MRT* were shortened, and the *CL* was decreased significantly in the serum of mice in MAFLD compared with the control group. The detailed pharmacokinetic parameters are shown in Table 5.

### 3.3. Enzyme Kinetic Parameters

**Figure 5:**
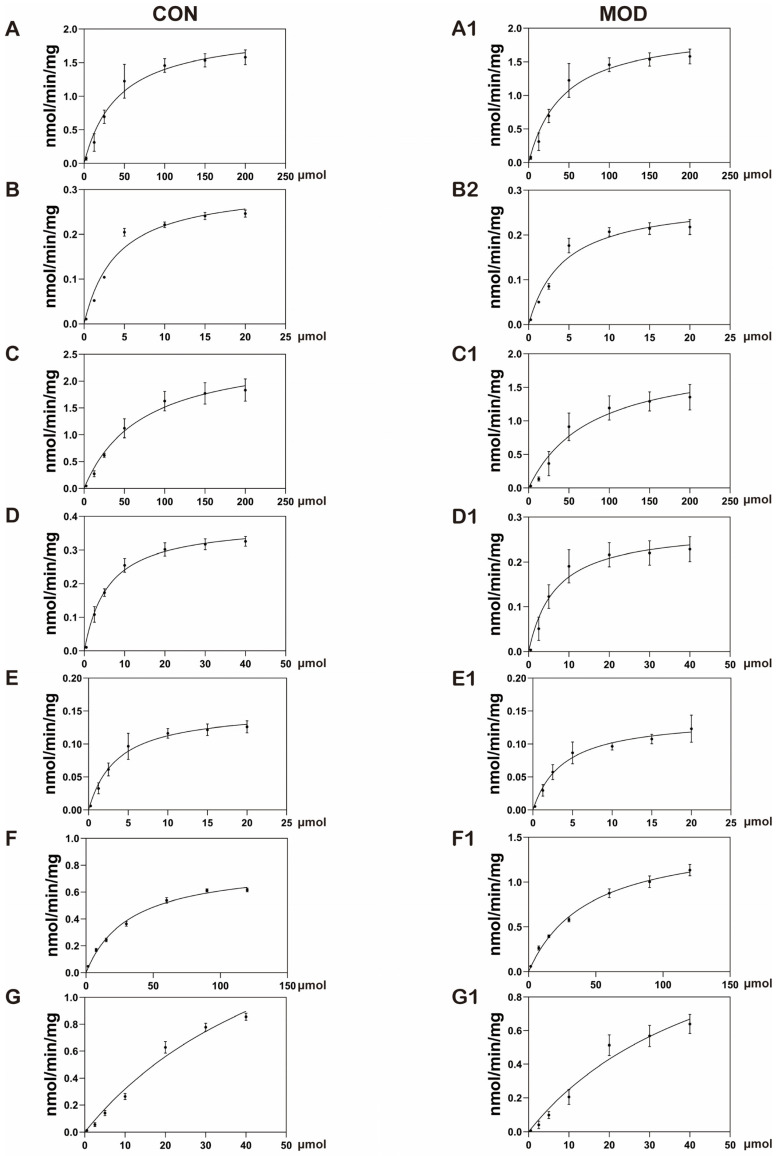
Probe substrate concentration C versus reaction rate V curve (*n* = 3). Normal liver cells, (**A**–**G**), MAFLD liver cells, (**A1**–**G1**). (**A**): Bupropion, (**B**): phenacetin, (**C**): testosterone, (**D**): chlorzoxazone, (**E**): diclofenac, (**F**): coumarin, (**G**): dextromethorphan.

The mother liquor prepared, as described in Section 2.4.1, was diluted with acetonitrile and then added to the liver cell incubation system. After preincubation at 37 °C for 5 min, 24 μL NADPH was added to the system to initiate the reaction. The system continued to incubate for 30 min; then, ice acetonitrile containing the internal standard was added to terminate the reaction. The system was vortexed and then centrifuged to obtain the supernatant. The supernatant was loaded to measure the remaining amount of substrates. Using the substrate consumption method and GraphPad Prism 8 software, the initial substrate concentration was plotted against the substrate consumption rate (Figure 5), and the *V_max_* and *K_m_* of each CYP450 enzyme subtype were obtained, as shown in Table 6.

#### Enzyme Kinetics Investigation

**Figure 6:**
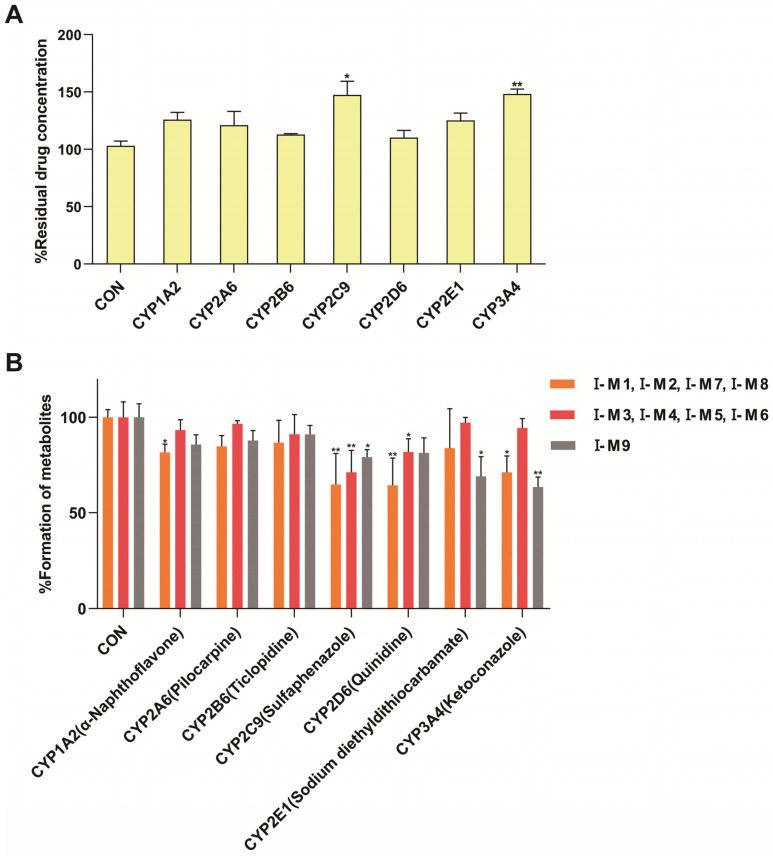
Formation of metabolites I-M1~I-M9 via the incubation of STB with liver microsomal enzymes (*n* = 3). Remaining amount of STB in the presence of various chemical inhibitors (**A**). Effects of selective CYP450 enzyme inhibitors on all metabolites in liver microsomes (**B**). * *p* < 0.05, ** *p* < 0.01.

**Figure 7:**
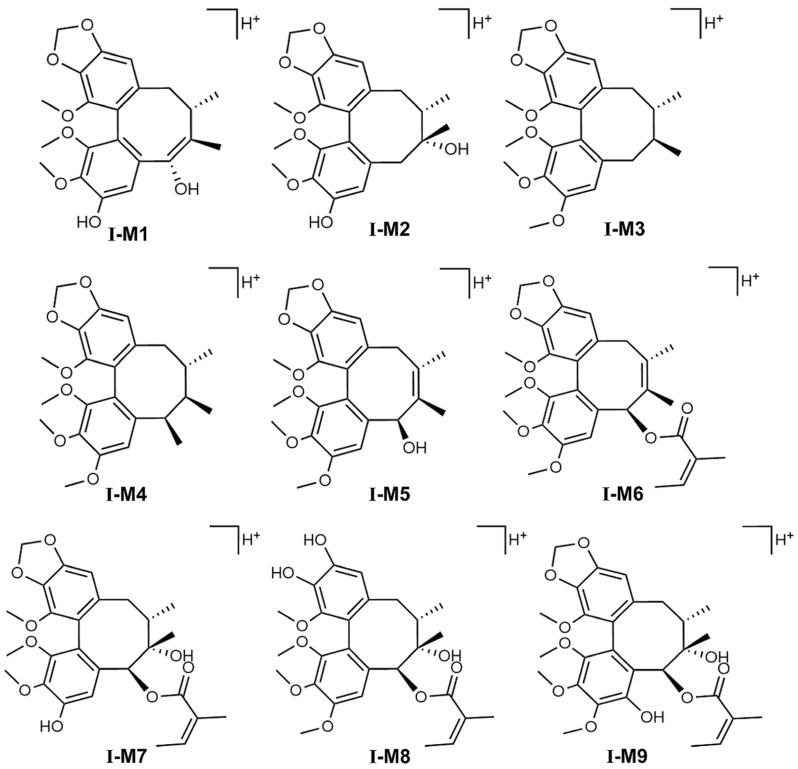
Structural formulas of metabolites identified in the culture of liver microsomes in mice.

The phenotype identification of STB was determined using a mixture of normal C57 mouse liver microsomal enzymes and selective chemical inhibitors. The conversion rate of major metabolites was monitored to evaluate the major enzyme subtypes involved in the metabolism of STB. The results showed that CYP3A4 and CYP2C9 were the main enzyme subtypes responsible for the formation of hydroxylated metabolites I-M9 and also the main pathways for the metabolism of STB in vivo (Figure 6A); CYP3A4, CYP2D6, CYP2C9, and CYP1A2 were the main enzyme subtypes responsible for the formation of demethylated metabolites I-M1, I-M2, I-M7, and I-M8; CYP2C9 and CYP2D6 were the main enzyme subtypes responsible for the formation of other reaction metabolites I-M3, I-M4, I-M5, and I-M6 (Figure 6B). Referring to our previous research [16,17] and related studies [23], the possible structures speculated for each metabolite are shown in Figure 7.

### 3.4. Metabolic Analysis of STB

#### 3.4.1. STB Mass Spectrometry Behavior

**Figure 8:**
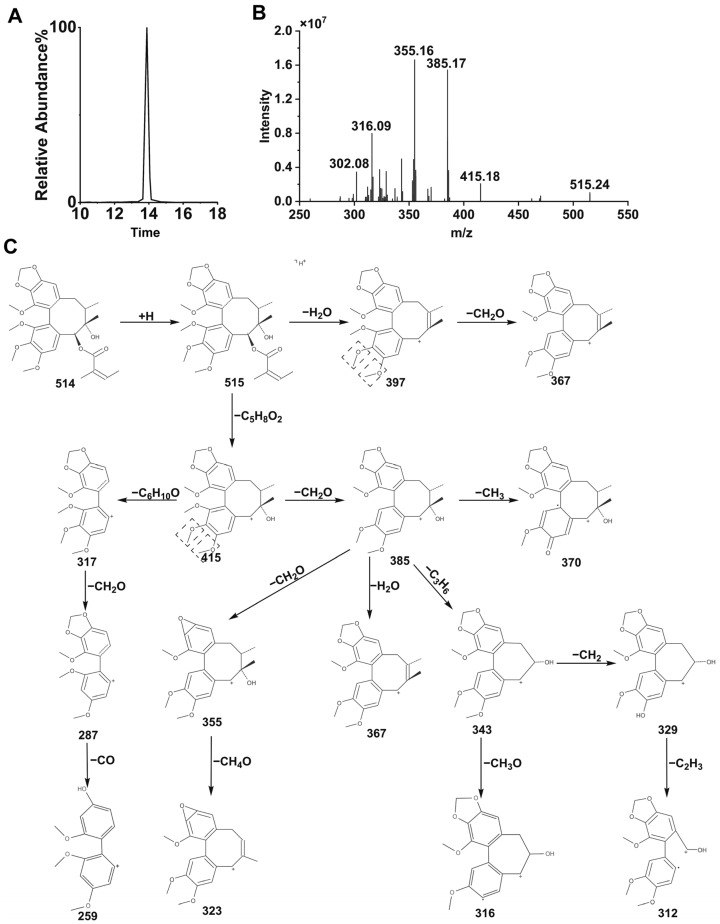
Extracted ion chromatogram (**A**), MS/MS spectrometry of STB (**B**), and fracture flow chart of STB under HPLC-ESI-MS/MS conditions (**C**). Dotted square: the potential demethylation site.

As shown in Figure 8, the retention time of STB was 12.93 min, and the molecular ions in HPLC-ESI-MS secondary mass spectrometry were [M+H]^+^ (*m*/*z* 515) and [M+H−C_5_H_8_O_2_]^+^ (*m*/*z* 415), indicating that STB is prone to losing one molecule of C_5_H_8_O_2_ (100 Da) at the C-6 position, the [M+H−C_5_H_8_O_2_]^+^ ion undergoes fragmentation under ESI-MS/MS to produce a series of fragment ions, *m*/*z* 397 is generated by losing one molecule of OH at the C-7 position, *m*/*z* 385 is generated by losing one molecule of CHO at the C-1 position, *m*/*z* 373 is generated by ring constriction and C_3_H_6_ loss at the octahedral ring, and *m*/*z* 371 is generated by losing a C_2_H_2_O molecule at the C-7 position. The fragmentation process of STB is shown in Figure 8C.

#### 3.4.2. Analysis of STB Metabolites

After oral administration of STB, due to the presence of various compounds in the blood and liver tissue of mice, HPLC-ESI-MS metabolomics technology was used to analyze the samples. SIMCA 14.1 software (Umetrics, Kinnelon, NJ, USA) was used for multivariate data analysis, mainly conducting principal component analysis (PCA), orthogonal partial least squares discrimination analysis (OPLS-DA), and s-plot score analysis. The criterion for screening potential metabolites was variable importance in projection (VIP) > 1.

**Figure 9:**
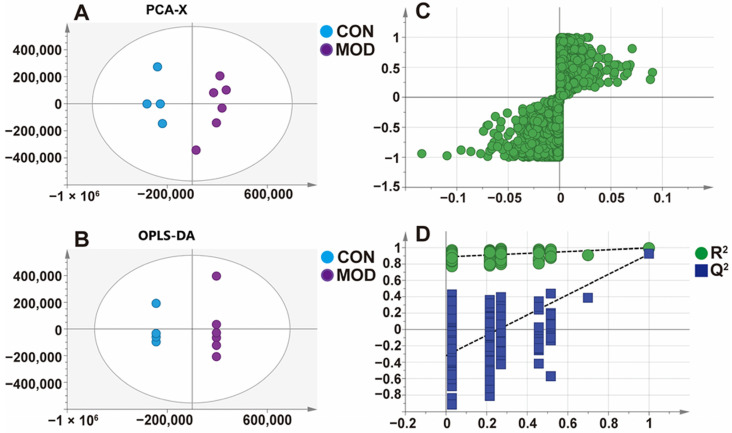
PCA score plot (**A**), OPLS-DA score plot (**B**), S-plot plot (**C**), and permutation testing of OPLS-DA model (**D**) of CON and MOD mice in positive ion mode.

**Figure 10:**
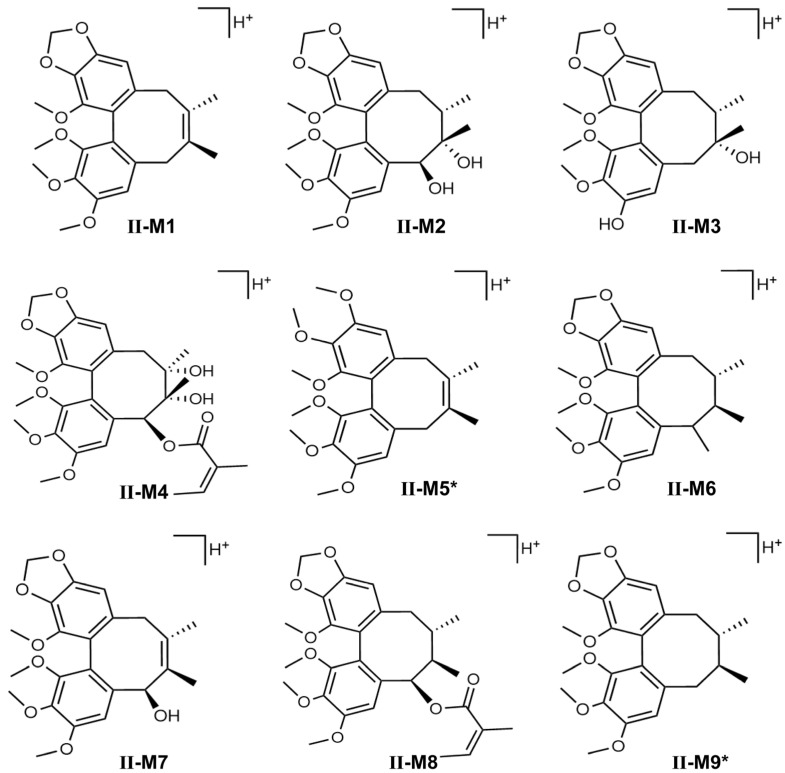
Chemical formulas of STB metabolites in mice (*: similar molecular weight and putative structure).

Taking the liver samples as an example, PCA analysis was used to perform matrix analysis on the control group (CON) and MAFLD model (MOD) sample data after oral administration of STB for 1 h. PCA, a commonly used unsupervised multivariate data analysis method, is considered to reduce the number of dimensions with minimum information loss and can show the characteristics of each sample. The PCA score plot (Figure 9A) showed a good separation between the two groups of samples. To further validate this, supervised OPLS discriminant analysis was used to maximize the difference of metabolites (unknown grouped samples). The OPLS-DA score plot (Figure 9B) was obtained by analyzing the data of the two groups, and the results showed that there was indeed a significant difference between the two groups of samples. The external model validation method (Figure 9D) was used to evaluate the effectiveness of the model. The results showed that the two regression lines presented a large slope, the R^2^ and Q^2^ generated by the left end arrangement were smaller than those of the right end, and the two values at the rightmost end were relatively close, demonstrating the effectiveness of the model. At the same time, S-plot analysis was conducted (Figure 9C) to further determine the metabolites of STB in the liver. Characteristic ions far from the origin were selected as potential metabolic markers, and Compound Discoverer 3.3 and Mass Frontier 8.0 software were used to analyze the precise molecular weight and fragmentation ion information of MS/MS. Finally, one STB prototype drug and nine metabolites were identified in the blood and liver tissue of mice after oral administration of STB (Figure 10, Table 7).

**Figure 11:**
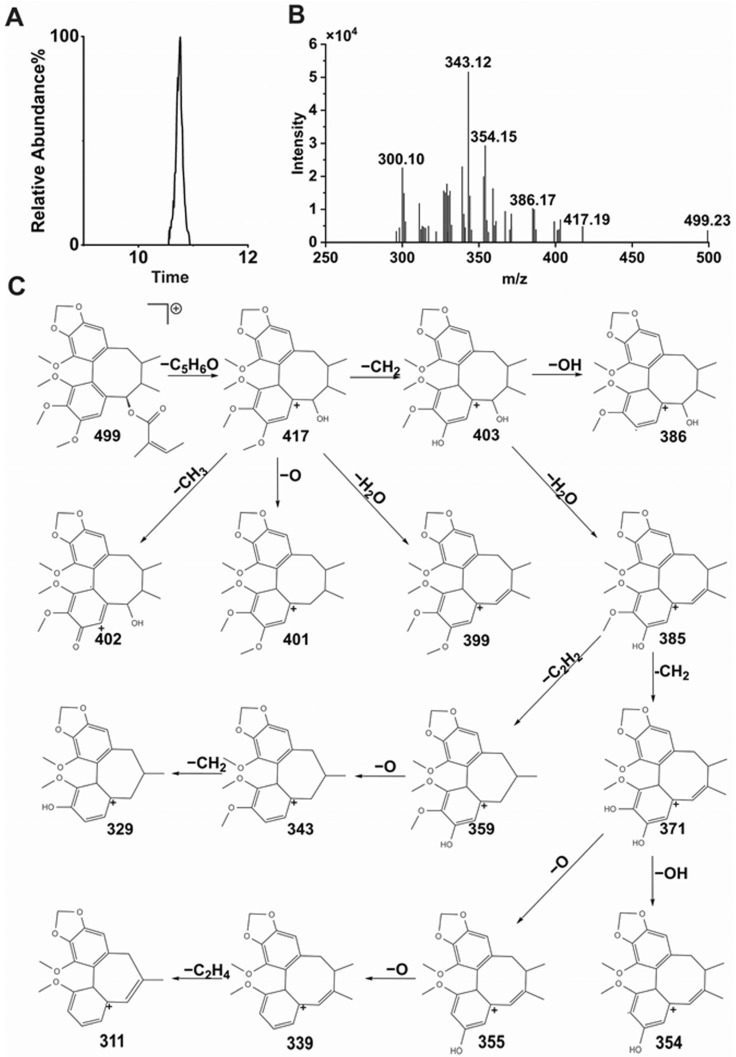
Extracted ion chromatogram (**A**), MS/MS spectrometry of STB (**B**), and fracture flow chart of M7 under HPLC-ESI-MS/MS conditions (**C**).

Taking metabolite II-M7 as an example, the metabolic mode of the original drug and the fragmentation process of metabolites was analyzed to identify nine metabolites. The retention time of II-M7 was 10.783 min (Figure 11A), and the mass to charge ratio *m*/*z* was 499.23192 (Figure 11B). In positive ion mode, the compound composition corresponding to *m*/*z* 499 was C_28_H_34_O_8_. Based on the chemical structural formula of the parent drug STB, the information obtained from the tandem mass spectrometry was decoded, speculating that the *m*/*z* 499 ion may be generated due to the loss of one hydroxyl group at the C-8 position of STB, *m*/*z* 499 may lose one molecule of C_5_H_6_O (82 Da) to generate fragment ions at 417, *m*/*z* 403 and 386 may be generated due to the loss of one molecule of CH_2_ and one molecule of methoxy (31 Da) at the C-12 position of *m*/*z* 417, respectively, *m*/*z* 385 may be generated by losing one molecule of H_2_O on its octatomic ring of *m*/*z* 403, *m*/*z* 359 may be generated by the condensation of one molecule of acetylene on its octatomic ring, *m*/*z* 371 and 354 may be generated by losing one molecule of CH_2_ and one molecule of methoxy (Da) at the C-13 position of *m*/*z* 385, respectively, *m*/*z* 355 may be generated by the loss of one molecule of oxygen at the C-13 position of *m*/*z* 371, *m*/*z* 339 may be generated by the further loss of one molecule of oxygen at the C-14 position of *m*/*z* 355, and *m*/*z* 311 may be generated by the condensation of one molecule of ethylene at the octahedral ring of *m*/*z* 339, as shown in Figure 11 C. The other MS/MS spectrometry data of II-M1-9 shown in Supplementary Material (Figures S1–S8).

It was found in the study of the metabolites of STB in MAFLD mice that the prototype drug of STB could be detected in both the blood and liver tissue of mice, metabolites II-M1~II-M9 could be detected in the liver tissue of normal mice, while only II-M6~II-M9 could not be detected in their blood. Seven metabolites II-M1~II-M7 were detected in the liver tissue of MAFLD mice, and only II-M4~II-M5 could not be detected in their blood.

## 4. Discussion

In this study, a MAFLD model was successfully established by feeding mice with a high-fat diet for induction. The model mice exhibited lipid metabolism disorders, with significantly increased levels of the serum AST, ALT, TC, TG, and LDL-C, significantly decreased levels of the serum HDL-C, and a significant hepatic steatosis. AST and ALT are often used as sensitive indicators of liver injury in clinical practice [24]. Fatty liver disease is closely related to lipid metabolism. Due to the reduced ability of the liver to metabolize fat, free fatty acids enter the body and cannot be metabolized normally, leading to an increase in circulating levels of TC, TG, and LDL-C and a decrease in HDL-C.

The in vitro incubation of AML-12 cells showed that multiple changes in cytochrome P450 enzymes could be observed in MAFLD model mice, including a 23% decrease in CYP1A2 activity, a 24% decrease in CYP2B6 activity, a 26% decrease in CYP2C9 activity, and a 33% decrease in CYP3A4 activity. There was no significant change in the activity of CYP2A6 and CYP2D6, while CYP2E1 activity increased nearly two-fold. A study analyzing liver biopsy samples from MAFLD patients showed a 30% decrease in CYP3A4 activity during the simple fatty lesion stage and a 50% decrease during the NASH stage, while the CYP2E1 activity increased by 2.5 times during the NASH stage [25]. Another study on the free fatty acid-induced MAFLD model of human liver cells also showed similar results, in which a 30–50% decrease in the activity of CYP1A2 and CYP2C9 was found [26]. The reasons for the changes in the activity of cytochrome P450 enzymes in the MAFLD model are shown in Table 8.

The study on pharmacokinetic characteristics revealed significant differences in the pharmacokinetic characteristics of STB between normal and MAFLD mice. Based on the bioequivalence criteria, the significant difference in drug exposure in MAFLD patients is defined as a 20% increase or decrease in the mean *AUC* in the model group compared to the control group [33]. In this study, the *AUC* of STB in the liver tissue of MAFLD mice was significantly reduced (the mean *AUC* was decreased by 40.2%), *C_max_* was significantly decreased, *t*_1/2_ was prolonged, *T_max_* and *MRT* were shortened, and *CL* was increased. The results showed that the total concentration of STB in the liver of MAFLD mice decreased, which is likely due to the occurrence of fatty lesions in the liver, leading to a decrease in microvascular blood flow and total effective liver cells, further resulting in a relative decrease in effective blood flow to the liver, ultimately reducing the exposure of STB in the liver of MAFLD mice. MAFLD is often accompanied by increased intestinal permeability and gastrointestinal motility abnormalities, which may lead to a reduced drug absorption. The increase in *CL* and the prolongation of *t*_1/2_ may be due to the effect of changes in metabolic enzyme activity and hepatic blood flow on the metabolism and excretion of STB.

Contrary to the results in the liver, the *AUC* of STB in the blood of MAFLD mice significantly increased (the mean *AUC* increased by 42.58%), the *C_max_* increased, *t*_1/2_, *T_max_*, and *MRT* were shortened, and *CL* decreased, which may be likely due to the decreased activity of liver microsomal enzymes in MAFLD mice, leading to a decrease in STB metabolism and an increase in its content. An increase in *C_max_* and a decrease in *T_max_* indicate an accelerated absorption of STB in the intestine or a weakened first pass effect. A significant increase in *AUC* and a decrease in *CL* may indicate impaired liver metabolism or bile excretion disorders. Xie et al. also successfully established a MAFLD model by administering a high-fat diet to rats and studied the pharmacokinetic behavior of lovastatin in MAFLD rats. The experimental results showed that the exposure of lovastatin in the plasma of MAFLD rats increased. After analyzing the reasons, the researchers believe that the main metabolic enzyme of lovastatin is CYP3A2. The pathological state of liver injury may lead to a decrease in the expression of this enzyme, thereby slowing down the metabolism of lovastatin, and the results are consistent with those reported in the related studies [34,35].

Drug-metabolizing enzymes in the body are mainly divided into phase I metabolizing enzymes and phase II metabolizing enzymes. The former mainly comprises oxidases, reducdases, and hydrolases, and the latter mainly comprises conjugated enzymes, of which the key enzyme systems involved in oxidative metabolism mainly include CYP, flavin monooxidase, monoamine oxidase, aldehyde dehydrogenase, ethanol dehydrogenase, aldehyde oxidase, and xanthine oxidase [36]. The existing research evidence suggests that the expression levels of drug-metabolizing enzymes (especially CYP) in the body are negatively correlated with drug exposure, and when CYP expression is downregulated, the systemic exposure of its metabolites may increase, while when its expression is upregulated, the substrate clearance accelerates, and the exposure decreases [37], consistent with our experimental results. Therefore, it is necessary to monitor these patients more closely in clinical practice to avoid the risk of toxicity [38]. In addition, free fatty acids in patients’ bodies can increase in MAFLD, leading to increased oxidative stress, which may result in the overexpression of CYP2E1 and CYP4A mRNA, as well as the increased activity of CYP2E1 [39,40]. Existing studies have identified multiple CYP subtypes involved in the metabolism of Schisandra chinensis lignans, mainly including CYP2C6, CYP2C11, CYP2E1, CYP3A, CYP1A2, and CYP2D2 [23,41,42,43], similar to the results that STB is mainly metabolized by the CYP3A4 and CYP2C9 enzymes.

In recent years, there has been an increasing amount of research on the in vivo metabolic pathways of *Schisandra chinensis* lignans. *Schisandra chinensis* lignans are mostly based on biphenyl cyclooctene as their skeleton structures, with side chains connecting different functional groups, and many studies have shown that substances with similar structures often have the same metabolic patterns. Zhang et al. pointed out that the metabolic pathways of STB in vivo are mainly demethoxylation, demethylation, and oxidation [23]. The results of this study also confirmed that STB was mainly metabolized in mice through several pathways such as demethoxylation, demethylation, and oxidation, consistent with the reported results.

It can be seen from the in vivo analysis of STB metabolites that its metabolites are mainly concentrated in the liver, with only a portion able to enter the bloodstream, which may reflect specific changes in liver metabolic activity and transport substances in MAFLD, such as the compensatory enhancement of liver metabolic enzyme activity, the obstruction of hepatic metabolite efflux, the accelerated clearance of metabolites in the blood, and differences in tissue distribution. It has been found that *Schisandra lignans* exhibits significant hepatic intestinal circulation characteristics in the body. Its metabolic process shows that a large amount of prototype drugs and their metabolites can enter the intestine with bile and undergo intestinal reabsorption or be excreted from the intestine. This circulation pattern may lead to a higher distribution of Schisandra lignans metabolites in liver tissue and a lower distribution in the blood. The metabolites of STB could be detected in normal mouse liver, while some of them were not detected in MAFLD mouse liver, which may be due to MAFLD affecting the distribution and metabolism of STB in mouse liver, thereby reducing its metabolites.

## 5. Conclusions

In vitro MAFLD cell incubation experiments showed that the activity of the CYP2C9, CYP3A4, and CYP2B6 subtypes was significantly reduced in MAFLD. The in vitro incubation experiment of liver microsomal enzymes showed that schisantherin B is mainly metabolized by the CYP3A4 and CYP2C9 subtypes. Therefore, changes in the activity of liver microsomal enzymes may cause changes in the metabolic rate and clearance rate of schisantherin B in MAFLD mice and then affect its distribution in the body.

## Figures and Tables

**Table 1:** Selective inhibitor concentrations.

CYP Isoform	Inhibitor	Final Concentration (mM)
CYP1A2	α-Naphthoflavone	5
CYP2A6	Pilocarpine	100
CYP2B6	Ticlopidine	10
CYP2C9	Sulfaphenazole	10
CYP2D6	Quinidine	5
CYP2E1	Sodium diethyldithiocarbamate	100
CYP3A4	Ketoconazole	1

**Table 2:** Metabolite gradient elution procedures.

Time (min)	Mobile Phases A (%)	Mobile Phases B (%)
0	65	60
5	65	60
12	45	40
25	15	10
28	10	10
30	65	60

**Table 3:** Probe substrate and specific metabolite gradient elution program.

Time (min)	Mobile Phases A	Mobile Phases B
0	98	2
15	25	75
15.2	5	95

**Table 4:** Cytochrome P450 substrates and internal standard (IS) parameters.

CYP Isoform	Substrate	Specific Reaction	Metabolite	Exact mass *(m*/*z)*	Ion Mode
CYP1A2	Phenacetin	O-Deethylation		180.10132	ESI^+^
			Acetaminophen	152.06990	ESI^+^
CYP2A6	Coumarin	7-Hydroxylation		147.04346	ESI^+^
			7-Hydroxycoumarin	163.13240	ESI^+^
CYP2B6	Bupropion	Hydroxylation		240.11401	ESI^+^
			6-Hydroxybupropion	238.14221 (-H_2_O)	ESI^+^
CYP2C9	Diclofenac	4-Hydroxylation		296.02280	ESI^+^
			4-Hydroxydiclofenac	312.35975	ESI^+^
CYP2D6	Dextromethorphan	O-Demethylation		272.19974	ESI^+^
			Dextrorphan	258.27814	ESI^+^
CYP2E1	Chlorzoxazone	6-Hydroxylation		167.98500	ESI^-^
			6-Hydroxychlorzoxazone	167.98506	ESI^-^
CYP3A4	Testosterone	6β-Hydroxylation		289.21512	ESI^+^
			6-Hydroxytestosterone	305.15625	ESI^+^
IS	Reserpine	-	-	609.38495	ESI^+^

**Table 5:** Primary pharmacokinetic parameters of STB in normal and MAFLD mice.

Pharmacokinetic Parameters	Blood		Liver	
	CON	MOD	CON	MOD
*T_max_* (h)	2.36 ± 0.32	2.16 ± 0.24	3.10 ± 0.35	3.06 ± 0.13
*C_max_* (μg·mL^−1^)	22.66 ± 1.64	25.81 ± 2.95 *	32.98 ± 3.60	19.74 ± 2.03 *
*t*_1/2_ (h)	7.49 ± 0.47	4.63 ± 0.21 *	5.34 ± 1.59	8.92 ± 2.12 *
*AUC*_0~_*_t_* (μg·mL^−1^·h^−1^)	186.32 ± 15.73	265.66 ± 23.51 *	425.09 ± 32.36	254.20 ± 26.33 **
*MRT*_0~_*_t_* (h)	15.42 ± 0.50	10.32 ± 0.22 *	8.04 ± 1.62	7.83 ± 0.96
CL (mL·h^−1^·kg^−1^)	15.36 ± 0.36	10.32 ± 0.09 *	11.25 ± 0.23	14.16 ± 1.06 *

* *p* ˂ 0.05, ** *p* ˂ 0.01.

**Table 6:** Enzyme kinetic parameters of normal liver cells and MAFLD liver cells.

CYP Isoform	*V_max_*			*K_m_*	
	CON	MOD	Compare	CON	MOD
CYP1A2	2.64	2.01 *	−23%	54.55	43.23
CYP2A6	0.31	0.28	-	4.10	4.30
CYP2B6	2.61	1.98 **	−24%	71.88	78.04
CYP2C9	0.38	0.28 *	−26%	5.93	6.69
CYP2D6	0.15	0.14	-	3.61	3.88
CYP2E1	0.81	1.53 ***	+88.9%	32.95	44.66
CYP3A4	2.26	1.51 ***	−33%	60.80	50.26

Note: *n* = 3, * *p* ˂ 0.05, ** *p* ˂ 0.01, *** *p* ˂ 0.001.

**Table 7:** STB and metabolites in mice.

ID	Formula	Rt (min)	*m*/*z* [M+H]^+^	Error (ppm)	MS/MS Fragment
STB	C_28_H_34_O_9_	13.92	515.2268	1.96	515.2307, 385.1664, 355.1555, 343.1198, 329.1039, 316.0957
II-M1	C_23_H_26_O_6_	16.41	399.1797	1.94	399.1824, 384.1591, 368.1638, 357.1344, 337.1451, 331.1188, 300.1008, 316.0968
II-M2	C_23_H_28_O_8_	12.98	433.1900	1.93	433.1893, 415.1707, 397.1659, 384.1961, 371.1510, 356.1270, 340.1317, 329.1013, 300.1036
II-M3	C_22_H_26_O_7_	7.58	403.1762	2.72	403.1737, 385.1666, 386.1726, 371.1491, 359.1519, 354.1468, 343.1195, 339.1254, 329.1401, 311.1290, 300.1008
II-M4	C_28_H_34_O_10_	10.67	531.2208	−3.16	531.2209, 396.2165, 373.2000, 357.2051, 355.1898, 339.1946, 337.1784, 319.2294, 314.2127, 301.2174, 291.2339, 273.2228
II-M5	C_24_H_30_O_6_	11.17	415.2126	2.65	415.2159, 400.1892, 385.2009, 384.1989, 373.1670, 359.1498, 328.1323, 316.1301
II-M6	C_23_H_26_O_7_	12.97	415.1759	1.46	415.1759, 397.1624, 385.1661, 371.1511, 367.1551, 356.1267, 340.1320, 329.1036
II-M7	C_28_H_34_O_8_	10.78	499.2319	−1.45	499.2319, 417.1911, 403.1737, 386.1726, 367.1561, 359.1520, 354.1467, 343.1196, 329.1401, 311.1291, 300.1008
II-M8	C_23_H_28_O_6_	16.45	401.1970	2.89	401.1982, 386.1743, 371.1865, 370.1806, 340.1691, 331.1195, 315.1260, 300.1010, 286.0860, 273.1136
II-M9	C_24_H_30_O_6_	9.89	415.2126	2.81	415.2131, 400.1906, 384.1957, 371.1535, 366.1475, 346.1398, 332.1192, 296.1800

**Table 8:** Reasons for changes in the activity of CYP450 enzyme subtypes.

Enzyme Phenotype	Change	Possible Reasons	Supported References
CYP1A2	↓	Inhibition of inflammatory factors, changes in nuclear receptors, oxidative stress	[7,8]
CYP2B6	↓	Inhibition of inflammatory factors, changes in nuclear receptors	[7,8,27]
CYP2C9	↓	Inhibition of inflammatory factors, changes in nuclear receptors, oxidative stress	[7,28,29]
CYP3A4	↓	Inhibition of inflammatory factors, changes in nuclear receptors, oxidative stress	[8,28,30]
CYP2E1	↑	Substrate induction	[31,32]

## Data Availability

The LC-MS data presented in the study are deposited in the Metabolights repository, accession number MTBLS13232. The original contributions presented in this study are included in the article and Supplementary Material. Further inquiries can be directed to the corresponding author.

## Supplementary Materials

The following supporting information can be downloaded at https://www.mdpi.com/article/10.3390/metabo15120763/s1, Figure S1: MS/MS spectrometry of II-M1 (*m/z* 399); Figure S2: MS/MS spectrometry of II-M2 (*m/z* 433); Figure S3: MS/MS spectrometry of II-M3 (*m/z* 403); Figure S4: MS/MS spectrometry of II-M4 (*m/z* 531); Figure S5: MS/MS spectrometry of II-M5 (*m/z* 415); Figure S6: MS/MS spectrometry of II-M6 (*m/z* 415); Figure S7: MS/MS spectrometry of II-M8 (*m/z* 401); Figure S8: MS/MS spectrometry of II-M9 (*m/z* 415).

## Abbreviations

The following abbreviations are used in this manuscript:

STB	Schisantherin B
MAFLD	Metabolic-associated fatty liver disease
HPLC-ESI-MS	High-performance liquid chromatography-electrospray tandem mass spectrometry
CYP	Cytochrome P450
AUC	Area under the curve
Cmax	Peak concentration
t1/2	Half-life
MRT	Mean retention time
CL	Clearance
TC	Total cholesterol
TG	Triglycerides
LDL-C	Low-density lipoprotein cholesterol
HDL-C	High-density lipoprotein cholesterol
ALT	Alanine aminotransferase
AST	Aspartate aminotransferase
HFD	High-fat diet
OA	Oleic acid
PA	Palmitic acid
IS	Internal standard
RSD	Relative standard deviation
PCA	Principal component analysis
OPLS-DA	Orthogonal partial least squares discrimination analysis
VIP	Variable importance in projection

## References

[1] Pipitone R.M., Ciccioli C., Infantino G., La Mantia C., Parisi S., Tulone A., Pennisi G., Grimaudo S., Petta S. (2023). MAFLD: A multisystem disease. Ther. Adv. Endocrinol. Metab..

[2] Araújo A.R., Rosso N., Bedogni G., Tiribelli C., Bellentani S. (2018). Global epidemiology of non-alcoholic fatty liver disease/non-alcoholic steatohepatitis: What we need in the future. Liver Int..

[3] Russo S., Kwiatkowski M., Govorukhina N., Bischoff R., Melgert B.N. (2021). Meta-Inflammation and Metabolic Reprogramming of Macrophages in Diabetes and Obesity: The Importance of Metabolites. Front. Immunol..

[4] Lewis D.F. (2004). 57 varieties: The human cytochromes P450. Pharmacogenomics.

[5] Dixit V., Hariparsad N., Desai P., Unadkat J.D. (2007). In vitro LC-MS cocktail assays to simultaneously determine human cytochrome P450 activities. Biopharm. Drug Dispos..

[6] Guengerich F.P. (2008). Cytochrome p450 and chemical toxicology. Chem. Res. Toxicol..

[7] Leclercq I.A., Farrell G.C., Field J., Bell D.R., Gonzalez F.J., Robertson G.R. (2000). CYP2E1 and CYP4A as microsomal catalysts of lipid peroxides in murine nonalcoholic steatohepatitis. J. Clin. Investig..

[8] Donato M.T., Lahoz A., Jiménez N., Pérez G., Serralta A., Mir J., Castell J.V., Gómez-Lechón M.J. (2006). Potential impact of steatosis on cytochrome P450 enzymes of human hepatocytes isolated from fatty liver grafts. Drug Metab. Rev..

[9] Szopa A., Ekiert R., Ekiert H. (2017). Current knowledge of Schisandra chinensis (Turcz.) Baill. (Chinese magnolia vine) as a medicinal plant species: A review on the bioactive components, pharmacological properties, analytical and biotechnological studies. Phytochem. Rev..

[10] Sun J.H., Liu X., Cong L.X., Li H., Zhang C.Y., Chen J.G., Wang C.M. (2017). Metabolomics study of the therapeutic mechanism of Schisandra Chinensis lignans in diet-induced hyperlipidemia mice. Lipids Health Dis..

[11] Feng Y., Li H., Chen C., Lin H., Xu G., Li H., Wang C., Chen J., Sun J. (2021). Study on the Hepatoprotection of Schisandra chinensis Caulis Polysaccharides in Nonalcoholic Fatty Liver Disease in Rats Based on Metabolomics. Front. Pharmacol..

[12] Wang C.M., Yuan R.S., Zhuang W.Y., Sun J.H., Wu J.Y., Li H., Chen J.G. (2016). Schisandra polysaccharide inhibits hepatic lipid accumulation by downregulating expression of SREBPs in NAFLD mice. Lipids Health Dis..

[13] Wang K., Zeng Q.C., Wan J.C., Song Z.R., Zhang Z.H., Wang C.M., Li H., Chen J.G., Sun J.H. (2024). Effect of schisantherin B on blood lipid levels in hyperlipidemic mice. J. Beihua Univ. (Nat. Sci.).

[14] Le Couteur D.G., Fraser R., Hilmer S., Rivory L.P., McLean A.J. (2005). The hepatic sinusoid in aging and cirrhosis: Effects on hepatic substrate disposition and drug clearance. Clin. Pharmacokinet..

[15] Zhu Y., Chen L., He Y., Qin L., Tan D., Bai Z., Song Y., Wang Y.H. (2023). The alteration of drug metabolism enzymes and pharmacokinetic parameters in nonalcoholic fatty liver disease: Current animal models and clinical practice. Drug Metab. Rev..

[16] Chen C., Feng Y., Li H., Lin H., Jing S., Li H., Wang C., Chen J., Sun J. (2022). Pharmacokinetics and Main Metabolites of Anwulignan in Mice. Front. Pharmacol..

[17] Wang J., Jiang B., Shan Y., Wang X., Lv X., Mohamed J., Li H., Wang C., Chen J., Sun J. (2020). Metabolic mapping of Schisandra chinensis lignans and their metabolites in rats using a metabolomic approach based on HPLC with quadrupole time-of-flight MS/MS spectrometry. J. Sep. Sci..

[18] Winzell M.S., Ahrén B. (2004). The high-fat diet-fed mouse: A model for studying mechanisms and treatment of impaired glucose tolerance and type 2 diabetes. Diabetes.

[19] Gómez-Lechón M.J., Donato M.T., Martínez-Romero A., Jiménez N., Castell J.V., O’Connor J.E. (2007). A human hepatocellular in vitro model to investigate steatosis. Chem. Biol. Interact..

[20] Zhou H., Tong Z., McLeod J.F. (2004). “Cocktail” approaches and strategies in drug development: Valuable tool or flawed science?. J. Clin. Pharmacol..

[21] Feidt D.M., Klein K., Hofmann U., Riedmaier S., Knobeloch D., Thasler W.E., Weiss T.S., Schwab M., Zanger U.M. (2010). Profiling induction of cytochrome p450 enzyme activity by statins using a new liquid chromatography-tandem mass spectrometry cocktail assay in human hepatocytes. Drug Metab. Dispos..

[22] Hewitt N.J., Lechón M.J., Houston J.B., Hallifax D., Brown H.S., Maurel P., Kenna J.G., Gustavsson L., Lohmann C., Skonberg C. (2007). Primary hepatocytes: Current understanding of the regulation of metabolic enzymes and transporter proteins, and pharmaceutical practice for the use of hepatocytes in metabolism, enzyme induction, transporter, clearance, and hepatotoxicity studies. Drug Metab. Rev..

[23] Zhang H., Jiang Y., Wu J., Zheng C., Ran X., Li D., Huang M., Bi H. (2017). Metabolic mapping of Schisandra sphenanthera extract and its active lignans using a metabolomic approach based on ultra high performance liquid chromatography with high-resolution mass spectrometry. J. Sep. Sci..

[24] Yao Z., Liu X.C., Gu Y.E. (2014). Schisandra chinensis Baill, a Chinese medicinal herb, alleviates high-fat-diet-inducing non-alcoholic steatohepatitis in rats. Afr. J. Tradit. Complement. Altern. Med..

[25] Fisher C.D., Lickteig A.J., Augustine L.M., Ranger-Moore J., Jackson J.P., Ferguson S.S., Cherrington N.J. (2009). Hepatic cytochrome P450 enzyme alterations in humans with progressive stages of nonalcoholic fatty liver disease. Drug Metab. Dispos..

[26] Fartoux L., Chazouillères O., Wendum D., Poupon R., Serfaty L. (2005). Impact of steatosis on progression of fibrosis in patients with mild hepatitis C. Hepatology.

[27] Vuppugalla R., Mehvar R. (2004). Short-term inhibitory effects of nitric oxide on cytochrome P450-mediated drug metabolism: Time dependency and reversibility profiles in isolated perfused rat livers. Drug Metab. Dispos..

[28] Aitken A.E., Morgan E.T. (2007). Gene-specific effects of inflammatory cytokines on cytochrome P450 2C, 2B6 and 3A4 mRNA levels in human hepatocytes. Drug Metab. Dispos..

[29] Assenat E., Gerbal-Chaloin S., Larrey D., Saric J., Fabre J.M., Maurel P., Vilarem M.J., Pascussi J.M. (2004). Interleukin 1beta inhibits CAR-induced expression of hepatic genes involved in drug and bilirubin clearance. Hepatology.

[30] Jover R., Bort R., Gómez-Lechón M.J., Castell J.V. (2002). Down-regulation of human CYP3A4 by the inflammatory signal interleukin-6: Molecular mechanism and transcription factors involved. FASEB J..

[31] Abdelmegeed M.A., Banerjee A., Yoo S.H., Jang S., Gonzalez F.J., Song B.J. (2012). Critical role of cytochrome P450 2E1 (CYP2E1) in the development of high fat-induced non-alcoholic steatohepatitis. J. Hepatol..

[32] Aubert J., Begriche K., Knockaert L., Robin M.A., Fromenty B. (2011). Increased expression of cytochrome P450 2E1 in nonalcoholic fatty liver disease: Mechanisms and pathophysiological role. Clin. Res. Hepatol. Gastroenterol..

[33] Aruna T., Yadav C.V.B., Nagabhushanam M.V., Bonthagarala B., Reddy D.N., Ramakrishna G.J.A.P. (2018). Guidelines for bioavailability and bioequivalence studies: A review. Pharma Innov..

[34] Xie Y., Wang H., Feng D., Hao H.P., Wang G.G. (2013). Effect of curcumin on the pharmacokinetics of lovastatin in rats with non-alcoholic fatty liver disease. J. China Pharma Univ..

[35] Patoine D., Levac X., Pilote S., Drolet B., Simard C. (2013). Decreased CYP3A expression and activity in guinea pig models of diet-induced metabolic syndrome: Is fatty liver infiltration involved?. Drug Metab. Dispos..

[36] Yin Z.Y., Ai J.Y., Gao J.K., Lin X.Y., Lu F.P., Qin H.M., Mao S.H. (2025). From Tradition to Innovation: The Transition of P450 Enzyme Catalysis via Light-Driven Electron Transfer. ACS Catal..

[37] Jamwal R., Barlock B.J. (2020). Nonalcoholic Fatty Liver Disease (NAFLD) and Hepatic Cytochrome P450 (CYP) Enzymes. Pharmaceuticals.

[38] Newman E.M., Rowland A. (2022). A Physiologically Based Pharmacokinetic Model to Predict the Impact of Metabolic Changes Associated with Metabolic Associated Fatty Liver Disease on Drug Exposure. Int. J. Mol. Sci..

[39] Nakamuta M., Kohjima M., Morizono S., Kotoh K., Yoshimoto T., Miyagi I., Enjoji M. (2005). Evaluation of fatty acid metabolism-related gene expression in nonalcoholic fatty liver disease. Int. J. Mol. Med..

[40] Kohjima M., Enjoji M., Higuchi N., Kato M., Kotoh K., Yoshimoto T., Fujino T., Yada M., Yada R., Harada N. (2007). Re-evaluation of fatty acid metabolism-related gene expression in nonalcoholic fatty liver disease. Int. J. Mol. Med..

[41] Liang Y., Hao H., Xie L., Kang A., Xie T., Zheng X., Dai C., Hao K., Sheng L., Wang G. (2010). Development of a systematic approach to identify metabolites for herbal homologs based on liquid chromatography hybrid ion trap time-of-flight mass spectrometry: Gender-related difference in metabolism of Schisandra lignans in rats. Drug Metab. Dispos..

[42] Cui Y.Y., Wang M.Z. (1993). Aspects of schizandrin metabolism in vitro and in vivo. Eur. J. Drug Metab. Pharmacokinet..

[43] Wu R., Xiao Z., Zhang X., Liu F., Zhou W., Zhang Y. (2018). The Cytochrome P450-Mediated Metabolism Alternation of Four Effective Lignans From Schisandra chinensis in Carbon Tetrachloride-Intoxicated Rats and Patients With Advanced Hepatocellular Carcinoma. Front. Pharmacol..

