# Supplementary material for: Metabolic Characteristics of Schisantherin B in Mice with Metabolic-Associated Fatty Liver Disease
Source: Metabolites. 2025 Nov 25;15(12):763. doi: 10.3390/metabo15120763 (PMC12734463; doi:10.3390/metabo15120763)
Supplement: Supplementary file 1 [file metabolites-15-00763-s001.zip › metabolites-3971947-supplementary.pdf]

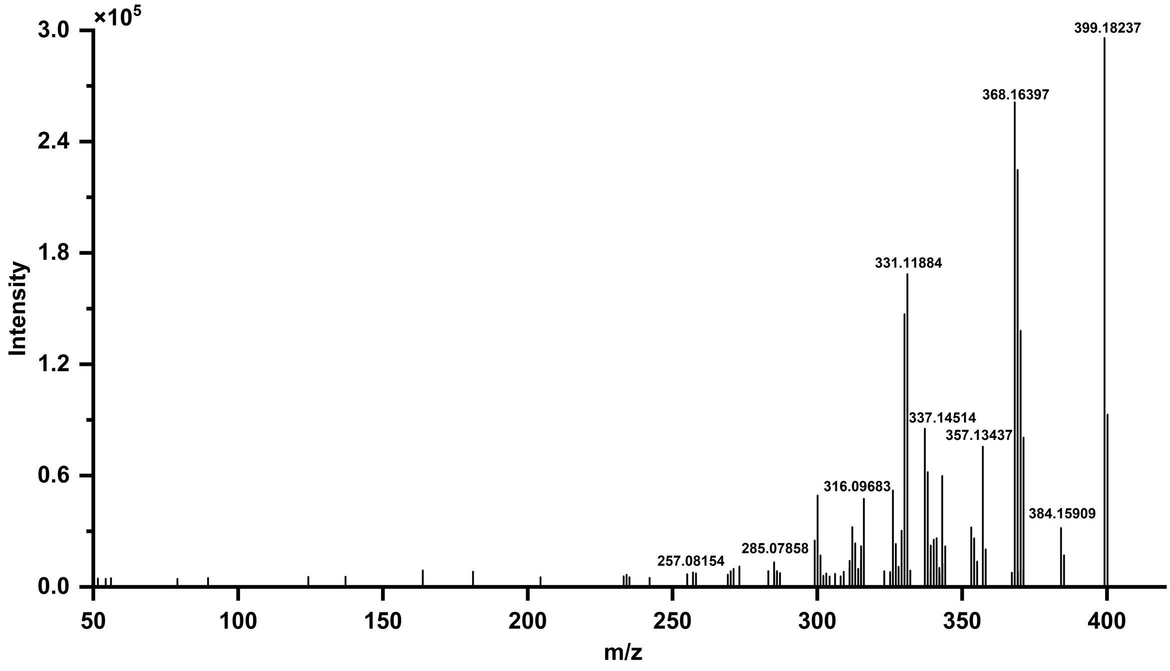

Supplementary Figure 1. MS/MS spectrometry of II-M1 (*m/z* 399)

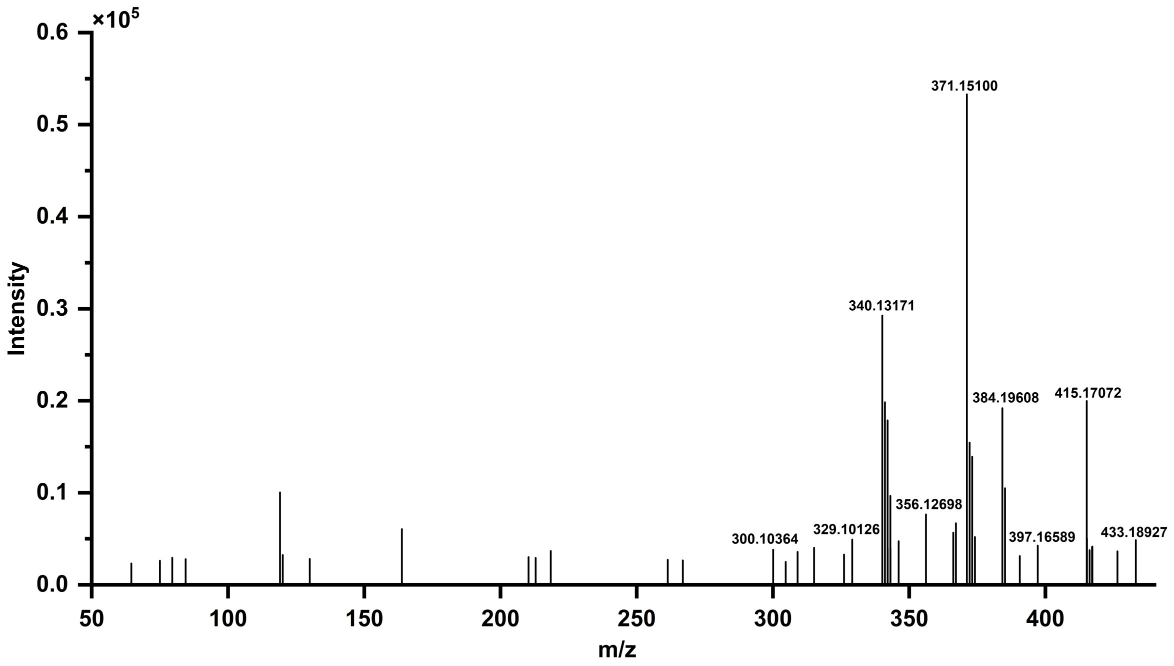

Supplementary Figure 2. MS/MS spectrometry of II-M2 (*m/z* 433)

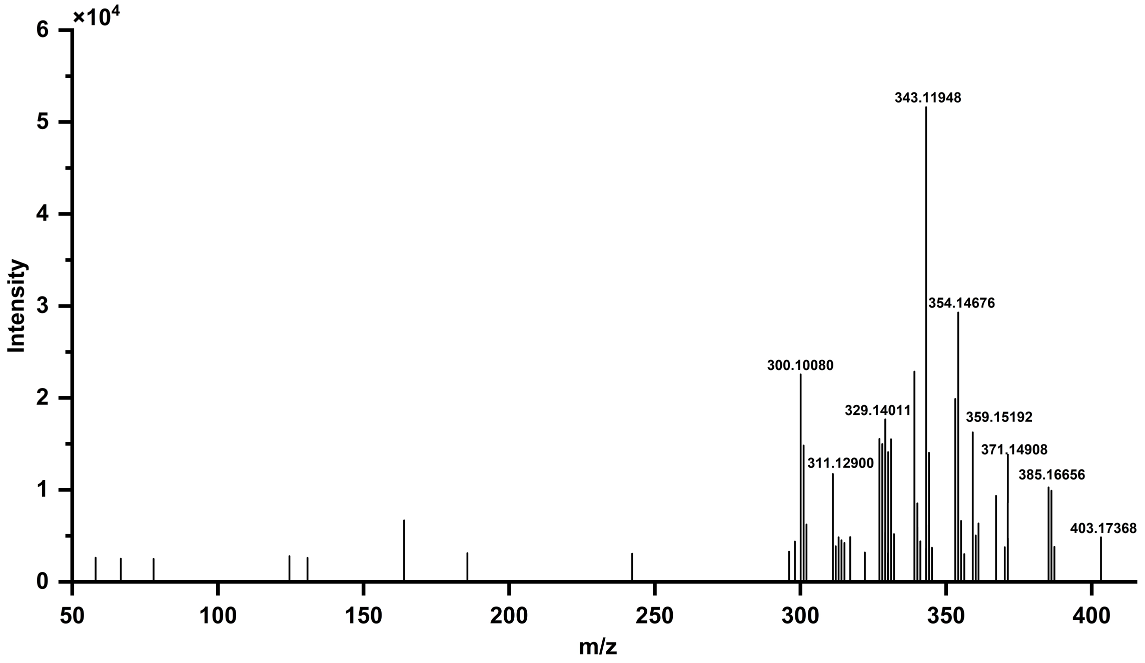

Supplementary Figure 3. MS/MS spectrometry of II-M3 (*m/z* 403)

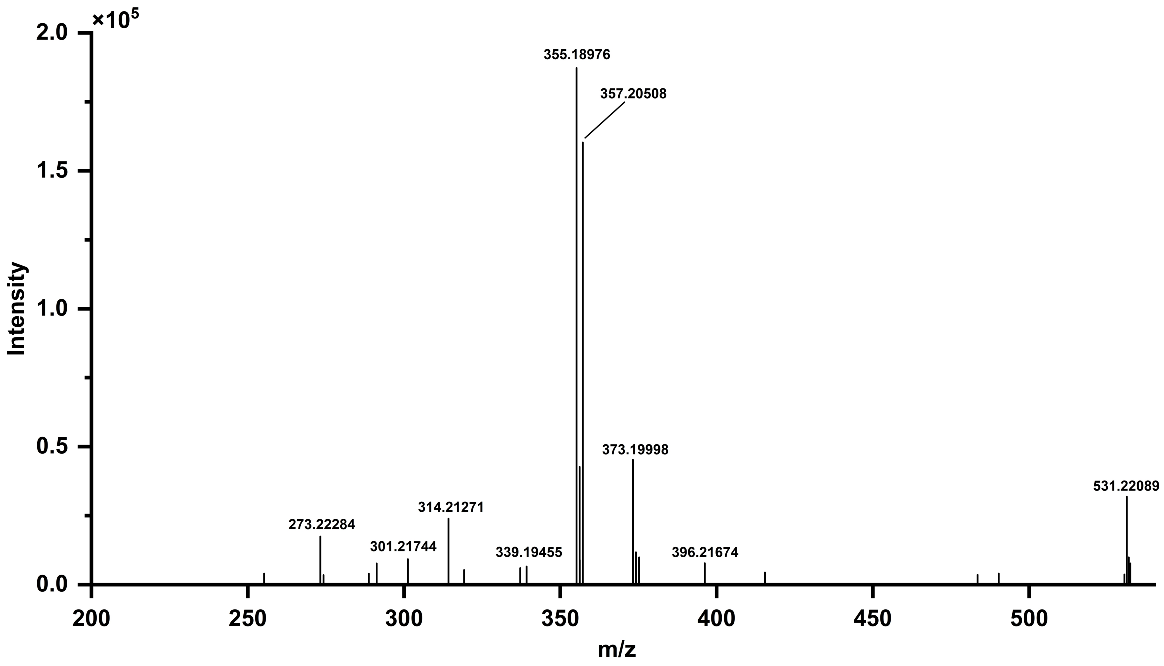

Supplementary Figure 4. MS/MS spectrometry of II-M4 (*m/z* 531)

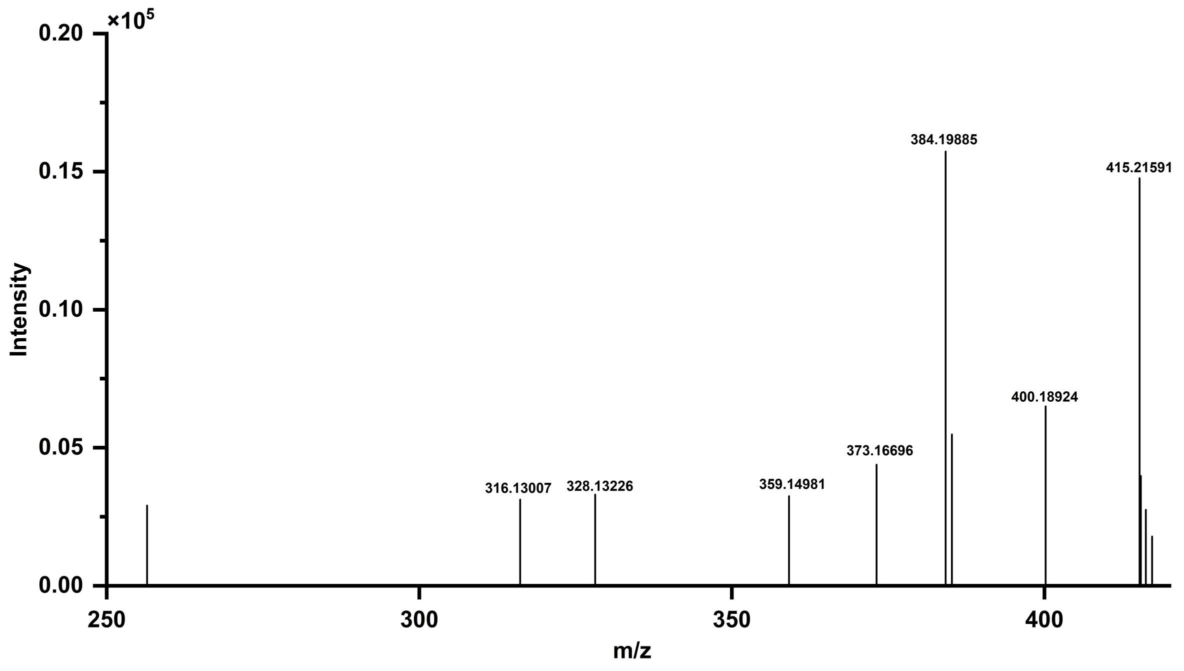

Supplementary Figure 5. MS/MS spectrometry of II-M5 (*m/z* 415)

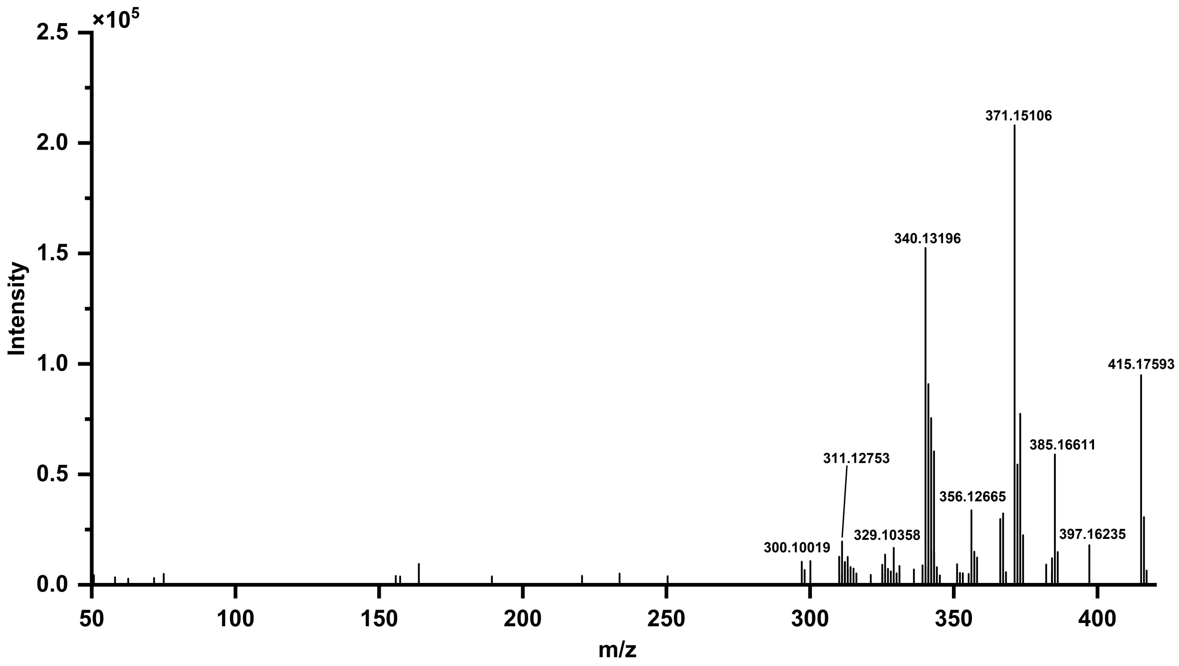

Supplementary Figure 6. MS/MS spectrometry of II-M6 (*m/z* 415)

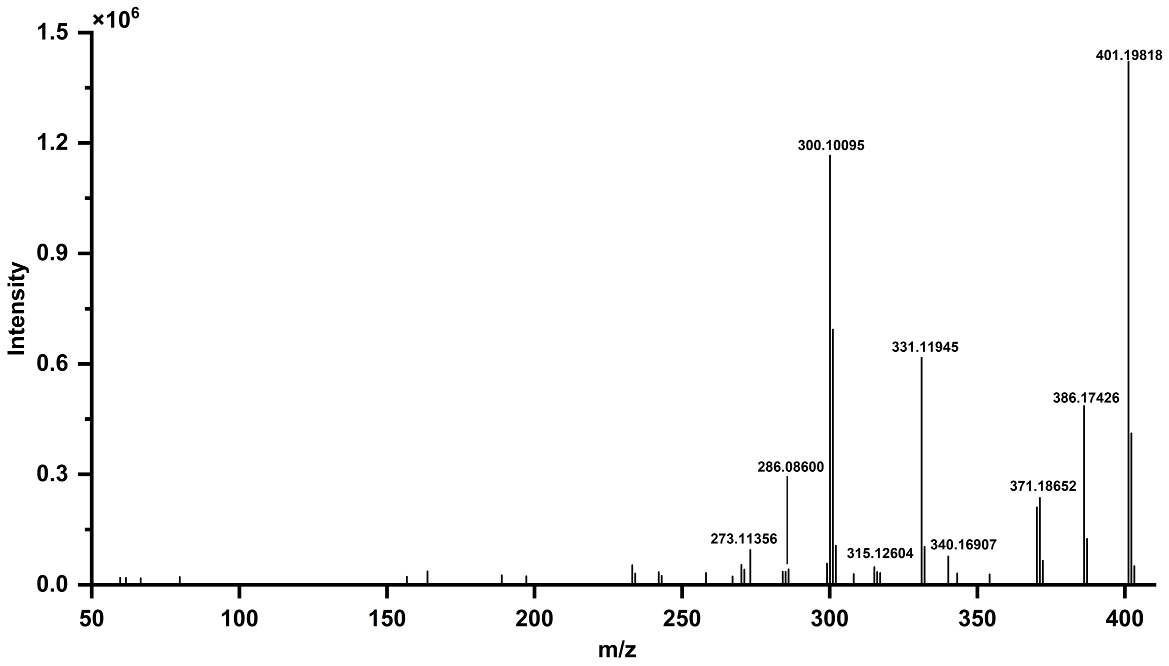

Supplementary Figure 7. MS/MS spectrometry of II-M8 (*m/z* 401)

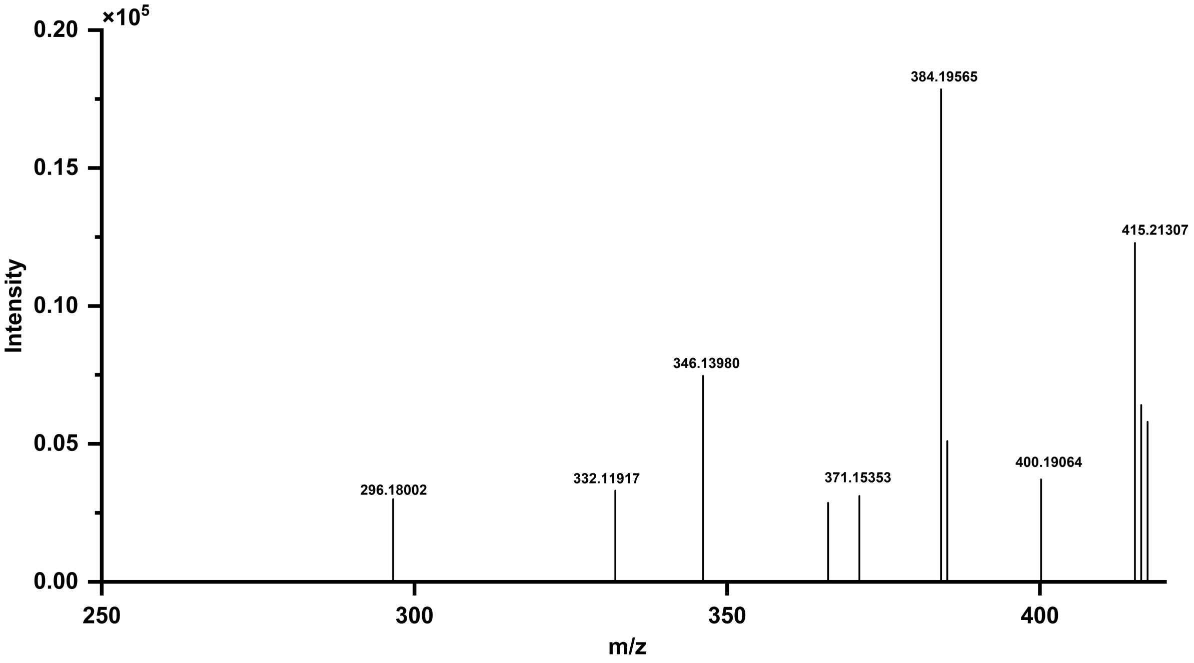

Supplementary Figure 8. MS/MS spectrometry of II-M9 (*m/z* 415)
